# A qualitative exploration of change processes relevant to compassion‐focused therapy that occurs when people view both soothing and non‐soothing images

**DOI:** 10.1111/papt.12566

**Published:** 2025-01-20

**Authors:** Stephanie Allan, Chris Morea, Keren MacLennan, Matthias Schwannauer, Angela L. McLaughlin, Netta Weinstein, Stella W. Y. Chan

**Affiliations:** ^1^ University of Glasgow Glasgow UK; ^2^ University of Reading Reading UK; ^3^ University of Durham Durham UK; ^4^ University of Edinburgh Edinburgh UK

**Keywords:** citizen science, compassion‐focused therapy, imagery

## Abstract

**Objective:**

Using soothing imagery within psychotherapy may support people to undertake positive visualisation exercises. However, little is known about what processes happen when people view images they find to be soothing or non‐soothing.

**Design:**

Exploratory qualitative methods were used.

**Method:**

Responses from 644 participants who had written about images they found to be soothing or non‐soothing were analysed using thematic analysis.

**Results:**

Two key themes were developed that related to the importance of the image content (such as it being a natural scene or artificial) and the internal cognitive and psychological processes that it triggered within participants as being key drivers for an image being perceived as soothing or non‐soothing. This included recall of positive autobiographical memories and liking the image content. Conversely, negative autobiographical memories and disliking image content were associated when people viewed images they considered to be non‐soothing.

**Conclusions:**

Experiences of feeling soothed when viewing an image appear to be maintained by imagining positive sensory experiences that were associated with the image or linking the image to positive experiences from the participant's autobiographical memory. This has implications for the delivery of therapy using pre‐existing image sets and suggests there is a need to find out what images are most suited for people accessing services.

## INTRODUCTION

Compassion‐focused therapy (CFT) is underpinned by the tripartite model of affect regulation which posits that three affect processing systems activate when responding to our environment and internal states (Gilbert, 2014). Within CFT, the concept of ‘soothe’ is related to being in a state of rest and digest, threat is related to the ‘fight or flight’ response and the drive system relates to motivations and resource seeking. (Gilbert, 2024). CFT aims to balance the threat‐protection system and the drive system, addressing anxiety and excitement, by focusing on individuals' symptoms and difficulties in terms of self‐protection. This approach targets feelings of self‐criticism, shame, safety, connectedness and contentment, helping individuals better regulate emotions related to drive or threat. The development of self‐soothing is an important target within this approach. This is because self‐criticism, dependency and shame—which are focused for CFT—increase the vulnerability of individuals to some mental health diagnoses, including social anxiety, personality disorders, eating disorders, depression, psychosis and suicidal ideation (Campos et al., 2013; Kannan & Levitt, 2013; Werner et al., 2019). Within CFT one of the main mediating factors contributing to successful CFT is vividness of mental imagery.

Mental imagery is the process of perceptually representing an experience without the associated stimulus present and is a basic form of cognition central to navigation, memory and problem‐solving (Kosslyn, 1996). It has been shown to play a pivotal role in mental health problems and treatments (Pearson et al., 2015). Guided mental imagery has been associated with greater positive affect change than verbal processing of similar events, while verbal processing could also increase negative mood, indicating that mental imagery can offer greater therapeutic changes than therapeutic discussion alone (Holmes et al., 2008; Nelis et al., 2012). CFT utilises guided imagery as part of the intervention, and is efficacious in treating symptoms of depression, stress and anxiety, while increasing levels of perceived comfort and enhancing self‐confidence (Apóstolo & Kolcaba, 2009; Vadoa et al., 1997).

However, mental imagery can be negative and intrusive, and biased toward negative interpretation which maintains depressed moods (Holmes et al., 2009). Research has further illustrated marked individual differences in the general population regarding mental image vividness (Cui et al., 2007). Overall, mental imagery may play a critical role in both the development and treatment of disorders like depression (Holmes et al., 2016). This makes CFT ideal for some treatments but inaccessible to those who may struggle to generate mental imagery. A way to resolve this issue is to provide access to positive soothing imagery.

Underpinned by CFT, Project Soothe (www.projectsoothe.com) is a global Citizen Science project that has collected 800 soothing images submitted by members of the public. These images were intended to activate the ‘soothe’ affective system that was theoretically predicted to play an important role in emotional regulation (Gilbert, 2009). These images primarily depicted images of nature, aligning with research that has established a link between exposure to nature and well‐being (Oswald et al., 2020). Earlier evaluation work has shown that viewing Project Soothe images has a positive effect on mood, (MacLennan, Byrne, et al., 2024; MacLennan, Schwannauer, et al., 2024; Witten et al., 2023) which supports the assumption the images may activate the soothing system. However, no known studies have focused on how feelings of soothe are understood and maintained when viewing images ‘*in vivo*’. Given the unknowns, the aim of the study was to use an inductive qualitative method to describe how people report feelings of change when viewing soothing images and to uncover potential maintenance factors. Furthermore, to improve credibility, this study also explored how people responded to images they considered non‐soothing.

## METHODS

### Design

This was an exploratory secondary analysis of the qualitative data collected within a larger research programme of which the quantitative analysis has been published elsewhere (MacLennan, Schwannauer, et al., 2024). We have stated this for transparency because there was not an initial plan to conduct an exploratory qualitative analysis of this data.

### Procedure

This study used an existing corpus of data. The larger programme of study was intended to understand the characteristics of the images within the Project Soothe image bank and how they may influence mood. This was accomplished by asking participants questions relating to their current mood, having them view and evaluate a selection of Project Soothe images and record their mood change from viewing this selection. Findings based on the quantitative data have been reported in full (MacLennan, Byrne, et al., 2024). The data provided by the wider study aligned with the aims of this current study, therefore it was appropriate to use and analyse this data.

Full informed consent was given on an online consent form before participants proceeded to take part in the study. Participants were asked to evaluate a random selection of 25 photos from the Project Soothe image bank, and rate how (a) soothed, (b) anxious and (c) excited each image made them feel on a scale from 1 (‘you don't feel that emotion’) to 7 (‘you feel that emotion very much’). Following this, participants were asked to provide more in‐depth evaluations regarding the images—they were shown a numbered collage of the 25 images they had just viewed, and asked to provide answers to the following:

1. ‘Which image do you find particularly soothing? (If you have more than one in mind, just choose the first one that pops into your mind just now)’.
2. ‘Inspired by the image, please tell a brief story based on an experience or imagined scenario that you associate with feeling soothed’.
3. ‘Which image do you not find particularly soothing? (Again, if you have more than one in mind, just choose the first one that pops into your mind just now)’.
4. ‘Inspired by the image, please tell a brief story based on an experience or imagined scenario that you associate with feeling soothed’.

To be able to answer more in‐depth, participants responded in a text box with a little limit on character count. This provided the original study with rich, qualitative data, which prior to the current study had not been analysed.

### Ethics

This study obtained ethical approval from the Department of Clinical and Health Psychology Research Ethics Panel at the University of Edinburgh and the School of Psychology and Clinical Sciences Research Ethics Panel at the University of Reading.

### Participants

Participant data came from a larger study of responses from 1003 participants. However, for data integrity, we excluded responses from participants who left responses blank or answered the same for both soothing and non‐soothing images. This left responses from 644 participants (64.2%).

### Analysis

The data for soothing and non‐soothing images were analysed using inductive thematic analysis which is a flexible method which can be used in explorative research (Braun & Clarke, 2006). This analysis has six steps: (1) becoming familiar with the data, (2) generating initial codes—where descriptive codes were initially constructed, (3) developing initial themes, (4) reviewing themes, (5) defining themes and (6) writing the report. Thematic analysis was guided throughout by the research aims (to understand participant experiences of viewing both soothing and non‐soothing images) and was an iterative and reflexive process that involved comparing and contrasting codes both between and interviews to construct themes. After step 6, the themes reported were considered by SA to appropriately represent the results of the analytic process.

### Trustworthiness and rigour

Reflexivity is key to thematic analysis (Braun & Clarke, 2023) which involves a critical examination of what researchers bring to the research process and their interpretation of qualitative data and their relationship to the research process. Author CM an MSc student trained in qualitative research was motivated to understand how people described their responses to images that they considered to be soothing and non‐soothing. To improve rigour and trustworthiness author CM kept a reflexive journal which included notes on potential biases and how their own positionality may influence the interpretation of data. To further ensure the clarity of the reported themes, the final thematic framework developed by CM was read alongside the raw data and agreed by another team member (first author SA) who was also motivated to develop an understanding of what processes may unfold when people view images they consider to be soothing or non‐soothing.

## RESULTS

We included responses from 644 participants. Most of the participants were female (*N* = 502, 76.6%) and came from European countries (*N* = 457, 70%) followed by the United States (*N* = 197, 30.1%). The average age was 34.2 years with a standard deviation of 14.8, ranging from 13 to 79 years. We have reported the results in line with the relevant parts of the Consolidated criteria for reporting qualitative research (COREQ) (Tong et al., 2007) checklist which can be seen in Appendix S1. Demographics can be seen in Table 1.

**TABLE 1:** Demographics of sample analysed (*N* = 655).

Baseline characteristic	*N*	%
*Gender*		
Female	502	76.6
Male	134	20.5
Other	8	1.2
Transgender	6	0.9
Prefer not to say	6	0.8
*Country*		
United Kingdom	276	42.1
United States	197	30.1
Belgium	25	3.8
Netherlands	25	3.8
Germany	17	2.6
France	16	2.4
Canada	12	1.8
Australia	11	1.7
Greece	11	1.7
Ireland	7	1.1
Other	58	8.9

## FINDINGS

Two key themes were developed from the data which were Image Content and Potential Mechanisms For Why Images Were Perceived as Soothing or Not Soothing. The overarching theme of *Image Content* considers information which describes the impact of actual image content on participant perceptions. Whereas, the overarching theme of *Potential Mechanisms For Why Images Were Perceived as Soothing or Not Soothing* refers to content which starts to theorise why some images were considered to be soothing or not soothing. Each theme is comprised of multiple subthemes. For soothing and non‐soothing categories, themes were found to work in contrast to one another and these differences are described with exemplar quotes below for transparency.

### Image content

#### Nature (soothing) versus artificialness (non‐soothing)

One way in which participants judged images to be soothing or non‐soothing was to appraise the environment of the picture.

*Nature* was often featured as the main environment participants judged images to be most soothing. When feeling soothed, participants often made references to various natural elements, the outdoors or the natural landscape. For example ‘seeing beautiful flowers or roses or anything that is blossoming is very soothing. Just seeing life in a peaceful setting, like in a grassy field is soothing’. (Female, 24). ‘No human activity to ruin it all. Just me sitting on the bank, quietly. Smiling’. (Female, 49). This highlighted that for some participants nature would also mean a lack of people or society.

*Artificialness, on the contrary, made images not soothing*. For example, one participant described an image they found to be non‐soothing as having ‘nothing green, no trees, grass of whatsoever. Soothing to me is an environment with water and (green) nature…’ (Female, 41). While another said: ‘the space in the image is non‐natural, artificial, man‐made. It invokes, noise, crowdedness, and the spirit of competition ‐ overall, to me, the opposite of soothing’. (Female, 45).

Overall, some participants assessed the environments of the pictures to be soothing or not soothing via the presence of nature or artificialness. Generally, participants found images filled with elements of the natural world to be soothing, but artificial images to have non‐soothing properties. The image became a representation of the environment which, in turn, influenced how they understood the soothing image in front of them.

#### Seclusion (soothing) versus busyness (non‐soothing)

Another way in which participants judged images to be soothing or non‐soothing was to make an appraisal of the activity, setting or substance of the image, as exemplified in the following extract describing a soothing image: ‘Quiet, no people. Lots of space and water gently lapping. Soothing sounds. For me the spaciousness and lack of busyness is soothing. Reminds me of time spent alone by the sea’. (Female, 38).

Busyness, on the contrary, referred to much higher levels of activity and how busy an image was. The level of energy appeared to be important for some participants as ‘Too much energy in the picture […]’ (Male, 45) and ‘the quality of the pic is blurred and imparts a sense of urgency and motion. The artifact in the picture is also puzzling and stimulates questions’. (Female, 33) explicitly referred to the content/image itself as imparting a sense of busyness (urgency and motion), while displaying too much activity.

Overall, seclusion and busyness—the level of activity, setting or substance—that was perceived to be in the image appeared then to influence how people judged an image to be soothing, which is captured in the following extract: ‘It looks either too crowded or too deserted. Once inside you're trapped inside. The water contributes to being trapped inside. If it were a museum, it might be very nice and pleasant. I like museums. But it doesn't look very much like a museum somehow. Too unadorned’. (Male, 61). For soothing images, this level was generally low and away from normal life, whereas for non‐soothing images there was either too much or too little activity, too close to everyday life with an overall sense of imbalance.

#### Content appeal (soothing) versus content aversion (non‐soothing)

A further key subtheme related to image content was whether participants found the image content appealing, in the case of soothing images or aversive in the case of non‐soothing images.

One participant only referred to their appeal—‘I like cats’—and aversion to the content—‘I don't like sunsets’ (Male, 26) as reasons for judging images as being soothing or non‐soothing.

Occasionally, the appeal would be in reference to other themes, such as nature. One participant mentioned, ‘I love the mountains and being outdoors, hiking and things like that. So, seeing pictures of there, while sadly not being able to be there, is very soothing.’ (Male, 22). Although this extract refers to the landscape, the participant mentions their love of mountains, outdoors and activities relating to it, rather than being soothed by the landscape itself. It was seeing a picture of what they find appealing that brought them soothing.

Overall, content appeal and aversion highlighted an aspect of the image that the person assessed at a surface level as being liked or disliked in general. The image became a representation of a subject which is already appraised positively or negatively which, in turn, influenced how they understood the soothing image they were viewing.

### Potential mechanisms for why images were perceived as soothing or not soothing

#### Positive memory recall (soothing) versus negative memory recall (non‐soothing)

Positive Memory Recall is related to recollection and experiences; this recollection may relate to a positive autobiographic memory recalled, or a reminder of something individuals find soothing such as a specific song, painting or activity they enjoy doing. At a basic level, participants were simply reminded of good times, such as the participant who stated, ‘The water and beach is the most soothing to me because it always reminds me of happy memories’. (Female, 37). While another participant stated the image brought back ‘memory of my youth sitting on the beach just looking out onto the ocean with the sun setting. A time I didn't have responsibilities or feel rushed’.

Conversely, for non‐soothing images, the pictures presented resulted in the recall of negative autobiographic memories. For example, here a participant states how seeing an image of a bathtub reminded them of a difficult life event:

‘Unfortunately, my cousin drowned in a bathtub when I was a child, and I don't know why but as soon as I saw that picture it came into my head straight away and I thought of it. I don't always think of it when I see bathtubs, but I did this time’. (Female, 36).

Overall, positive and negative memory recollections elicited by the image caused some participants to access real‐life soothing or non‐soothing elements outside of the photo. These were in the form of specific memories, general events or practices which participants found soothing or non‐soothing in the real world. This, in turn, influenced how they understood the soothing imagery presented.

#### Pleasant sensory experience (soothing) versus negative sensory experiences (non‐soothing)

Beyond autobiographic memories, the images appeared to bring up embodied sensory and haptic ideas of being a person in the world. For images found to be soothing, participants seemed to envision or imagine some form of pleasant or calming experience, describing interactions they would have with the image beyond just their enjoyment of the landscape or the content in it. Sometimes, this was explicitly because of viewing the image: ‘By looking at the picture I can imagine a warm breeze and feel the sand on my feet’. (Female, 61). For other participants, these experiences would be described as direct interaction with the image, setting themselves as part of a soothing scene: ‘walking on a sunny beach ‐ makes me think of hearing the water and smelling the sea’. (Female, 29).

*Negative Sensory Experiences*, however, would involve extrapolated or imagined scenarios participants would interpret as somehow uncomfortable for the senses. For example, sound: ‘The Scenario must be very, very loud. Noise in general doesn't make me feel soothed at all’. (Female, 34), temperature: ‘A long cold walk, in humid weather ‐ no one is around’ (Female, 36) or physical discomfort such as, ‘Makes me think of getting my hand trapped in machinery’ (Female, 39).

#### Sense of inner peace (soothing) versus threat to peace (non‐soothing)

Participants sometimes showed they understand images as soothing by being made more aware of their internal states, particularly relating to the sense of quiescence and calm as described by Gilbert (2009). The theme of *Sense of Inner Peace* is related to being made aware and drawing upon inner states of calming. Further research into this definition matched the feelings described by participants and so this study related the sense of peace using definitions given by Wong and Bowers (2019) as a process of finding ‘mature happiness’ characterised by ‘self‐acceptance, inner serenity, contentment, and being at peace with oneself, others and the world’ (p.112). The feeling itself was defined as feelings of relaxation, a state of calm, safety and protection from stress (Hanson, 2016), as displayed by this participant's response: ‘The associated feelings with the image involve, soothing, calmness, relaxed, submerged in the view and forget all the worries’. (Female, 49). Overall, these images would highlight those internal states and judge them against the image, as opposed to simply recalling them. Other images would elicit a sense of connectedness ‘I am Welsh and therefore I identify with this image more. Makes me feel happy and calm, makes me feel closer to Wales’. (Female, 38); the identification to image promotes feeling a sense of closeness and connectedness seemed to bring this sense of inner peace.

*Threat to Peace* was in contrast to *Sense of Inner Peace*. This would involve any times participants felt anxiety, threatened or other perceptively negative internal states or feelings that would make these images the least soothing for them, thus their peace was threatened. For example, a participant mentioned that, as a result of looking at one image they were reminded that ‘being at a crowded beach or just being surrounded by others creates a sense of anxiety and an on‐edge feeling’. (Female, 18). The participant processed the image in such a way that evoked a ‘sense of anxiety and an on‐edge feeling’, unrelated to the general busyness of the picture but being made more aware of those inner feelings. Other participants processed threats to peace from awareness of peaceful states then drawing into the more threatening elements: ‘The idea being close to a waterfall does not soothe me at all, quite the contrary, I feel worried. I cannot relax, I am scared that I or someone I like could fall down and die’. (Female, 27).

Overall, senses of inner peace and threats to inner peace elicited by the image caused some participants to be more aware, conscious or attuned to feelings and emotions that brought them a state of calm, or alternatively a state of unease, which, in turn, influenced how they understood soothing images they saw.

### Ethical or empathy breach (non‐soothing)

This theme was unique to non‐soothing images. There was no contrast found with soothing images. *Ethical or Empathy Breach* was a consideration of participants' personal ethical rules or some sense of self which were violated or discomforted by the imagery they were reflecting on. Oftentimes this would go against personal views and bring them into challenge head‐on. This would bring a sense of discomfort or opposition to their sense of inner peace which participants would then envision rectifying: ‘As I looked upon the sad monkey, unable to be free as he was intended to be, I open the cage and let him run freely. I could almost see a smile and a wave of his hand as he went to his freedom’. (Female, 70). Here, the rules of freedom are opposed by the image of the monkey. The participant goes on to create a scenario in which this breach is resolved, however, this image remains non‐soothing as this rule has been challenged. The images would act as a marker to emulate real‐world scenarios which directly challenge a sense of self or sense of inner peace related to the wider world: ‘Reminds me of the flowers the old ladies who lived next to my primary school. You're not enough of a girl if you don't like the flowers, you're different […]’ (Female, 23).

## DISCUSSION

This study explored people's direct constructions and appraisals of images that they considered to be soothing or non‐soothing. Previous research has observed that small improvements in mood have been observed when people viewed Project Soothe images (MacLennan, Schwannauer, et al., 2024) but there has been little examination of what processes may underpin this effect. Participants described soothing images as ones that evoke a connection to the natural world, supporting previous research indicating that nature exposure improves well‐being (Oswald et al., 2020) and suggests that this effect may be driven in part by the activation of the soothe system. Furthermore, soothing images were described as containing content that appealed to viewers on a personal level which may be driven by activation of positive autobiographical memory associations that bring people into contact with their attachment system (Gilbert, 2017). In contrast, when viewing non‐soothing images, the thematic analysis revealed that these images may instead activate the threat system by violating the viewer's ethical or empathic values.

### Implications for clinical practice

There is a need for wider accessibility of therapies. Additionally, psychological therapies which use pre‐existing images may be more accessible for people who struggle to generate their own mental imagery. When applied to the psychotherapeutic practice of using pre‐existing soothing imagery within sessions, these findings speak to the importance of finding out clients' preferences for types of image content given that liking image content appeared as an important part of what makes an image soothing. Exploring how clients appraise the images and what memories they invoke appears important to ensure the images are appropriate for the treatment target of helping a person to develop a sense of soothe.

The harm experienced by clients can be overlooked in psychotherapy (Mc Glanaghy et al., 2022) These findings highlight the importance of working safely with clients during therapy. For example, while the role of autobiographical memories could be therapeutically meaningful if soothing images remind people of pleasant times, traumatic experiences could be triggered by images designed to be soothing. This emphasises the importance of exploring individuals' perceptions of images rather than assuming that pre‐validated images will be universally soothing.

### Implications for research

A key finding of this study is that nature images are perceived as soothing, and images with artificial content are typically non‐soothing. Urban areas have a higher prevalence of psychiatric conditions than rural, or more natural, areas (Peen et al., 2010). The role of nature, therefore, is often implicated in research as contributing to positive mental well‐being, improved cognitive function and decrease of physiological stress (Bratman et al., 2012, 2019). Due to the lack of greenery in urban areas, however, it can be difficult for urban residents to access these benefits (Mitchell et al., 2015). Therefore, imagery has been used to elicit the effects of greenery in place of real natural environments (Jo et al., 2019). Future research could empirically test whether using nature images increases a sense of soothe when working with clients compared to other image types.

### Limitations

This finding of the study must be considered in light of limitations. First, we did not assess the presence of mental health problems experienced by participants. While data from this study were important in developing a theory for understanding how a sense of soothe is developed when viewing images, it is currently unclear if these processes would apply within therapeutic encounters for people who experience mental health problems. Second, the participants mostly represent people from high‐income countries. Future research should include communities from the global south to examine whether any processes identified as underpinning images being perceived as soothing or non‐soothing could be considered universal.

### Considerations for future research

It would be beneficial to determine the extent to which these identified processes drive any observed positive mood changes after viewing soothing images (MacLennan, Schwannauer, et al., 2024) using techniques such as mediation analysis. We are cautiously optimistic that the thematic codes constructed in this study may be useful for use in automatic image generation using digital means such as artificial intelligence (AI). The study highlights the variability in what people find soothing and the potential importance of personalising therapeutic tools. From a practical standpoint, AI‐based customisation could offer a tool to address these individual differences, but it would need to be deployed with sensitivity to the complexities of preferences, personal history and sensory experiences. Therapists using such tools might need to remain vigilant about potential unintended effects and work closely with clients to ensure that the images support, rather than disrupt, therapeutic goals. Ongoing research will be needed to ensure these images provide genuine therapeutic benefits and avoid unintended negative effects.

In this study, we assumed that participants would understand ‘soothe’ in line with previous work showing that individuals understand their subjective experience of soothing as complex interconnected feelings, as a distinction between self‐soothing and being soothed, and complex interactions of physical sensations (Mok et al., 2019). However, 35.7% of the data were removed due to interpretations of ‘soothe’ that did not align with this definition (to the extent that rendered doubts as to whether participants were able to understand and follow the instructions of the study). Therefore, it may be that there are individual differences in the way soothe is interpreted and experienced. For instance, a participant reported that: ‘soothe is a term/description that I find very hard to relate to—feels pretentious and sound middle class’. Future work should critically engage with understanding individual differences in understanding the soothe concept. Connectedness was another theme relevant to understand how the images developed a sense of inner peace. Participants made reference to connectedness which could relate to familiarity with a place, community or a sense of cultural or ethnic identity. Future research should consider how the concept of soothe is conceptualised, and how images considered to be soothing are interpreted by people from around the world and across neurotypes.

## CONCLUSION

Project soothe is a long‐term citizen science project that invites people to submit images of what they find soothing and then validate the soothingness of these images externally. This study directly adds to our understanding of processes that underpin the activation of the soothing system when people view images they personally consider to be soothing, informing the development of a psychological understanding of these processes. Images appraised as soothing may contain content that is personally appealing, related to nature and depicts seclusion, as well as processes related to positive memory recall, pleasant sensory perceptions and a sense of inner peace. This work supports the use of externally presented soothing imagery to facilitate positive mental imagery in psychotherapies such as CFT.

## AUTHOR CONTRIBUTIONS

**Stephanie Allan:** Writing – original draft; writing – review and editing; formal analysis; validation. **Chris Morea:** Writing – original draft; formal analysis. **Keren MacLennan:** Writing – review and editing. **Matthias Schwannauer:** Conceptualization; investigation; funding acquisition; methodology; writing – review and editing; data curation. **Angela L. McLaughlin:** Writing – review and editing; project administration; data curation. **Netta Weinstein:** Writing – original draft; writing – review and editing. **Stella W. Y. Chan:** Conceptualization; investigation; funding acquisition; writing – original draft; methodology; writing – review and editing; supervision; data curation; project administration; formal analysis.

## FUNDING INFORMATION

NW's time on the project was supported by funding from the European Research Council (ERC: SOAR‐851890).

## CONFLICT OF INTEREST STATEMENT

The authors declare no conflicts of interest.

## Supporting information


Appendix S1

## Data Availability

The data that support the findings of this study are available on request from the corresponding author. The data are not publicly available due to privacy or ethical restrictions.
